# Sequence-controlled peptoid hydrogels for neuronal trans-differentiation: a synthetic biomaterials approach in future neuroregenerative research

**DOI:** 10.1039/d5md00610d

**Published:** 2025-09-29

**Authors:** Deviramma C. Yamanur, Pratikshya Paudel, Prabir Kumar Gharai

**Affiliations:** a Department of Physics, Indian Institute of Science Education and Research Tirupati AP 517507 India cydevi20@gmail.com; b Department of Chemistry, Oklahoma State University Stillwater OK 74078 USA pratikshya.paudel@okstate.edu; c Department of Animal and Food Sciences, Oklahoma State University Stillwater OK 74078 USA pgharai@okstate.edu pkgiicb@gmail.com +1 405 269 1764

## Abstract

Peptide-based hydrogels stimulate stem cell differentiation into neurons, improve neuroprotection, and aid in spinal cord/brain injury repair by moderating inflammatory responses and activating endogenous healing pathways. Peptoid-based hydrogels, with superior enzymatic stability and tunable properties, offer a promising alternative but remain unexplored in neurodegenerative therapies. Thus, future study should focus on optimizing peptoid-based hydrogel formulations, which could considerably progress neuroregenerative therapies in neuroscience research.

## Abstract 2

Transplanted stem cells, when supported by peptoid-based hydrogels, demonstrate enhanced migration to the injury site, improved survival rates, and facilitate brain repair through the formation of new connections and support for recovery.

## Graphical abstract

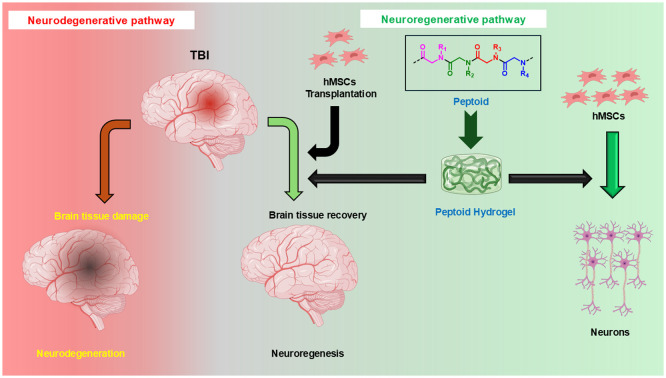

Traumatic brain injury (TBI) is still a prominent global cause of death and long-term disability.^1^ Despite decades of study into neuroprotective therapeutics targeted at protecting neural tissue during the acute phase of damage, few of the promising preclinical treatments have been translated into viable clinical interventions. TBI frequently requires lengthy medical care, resulting in significant economic and emotional consequences. TBI comprises two main phases: primary and secondary damage. The initial injury is caused by an immediate mechanical impact, which causes structural damage such as tissue distortion, vascular rupture, and necrotic cell death. This initial trauma causes a complicated secondary injury cascade that includes excitotoxicity, calcium dysregulation, inflammatory responses, metabolic dysfunction, oxidative stress, dysregulated autophagy, and programmed cell death. These secondary pathways amplify neuronal injury and contribute to long-term functional limitations. As a result, therapeutic techniques that target these delayed degenerative processes may provide more effective treatment for TBI patients.

Current therapies for neurological conditions generally provide symptomatic relief, emphasizing the critical need for disease-modifying medications. Among developing possibilities, stem cell-based therapies, particularly those utilizing mesenchymal stem cells (MSCs), hold great promise for reducing disease progression and boosting neurological recovery.^2^

MSCs are pluripotent, non-hematopoietic stem cells that can self-renew and differentiate into multiple lineages. They can be extracted from a variety of adult tissues, including the bone marrow, umbilical cord, adipose tissue, and amniotic fluid, and easily grown *in vitro* for application in regenerative medicine. MSCs have demonstrated therapeutic potential in a variety of disorders, including neurological disorders and injuries.^3^

Their extensive relevance arises largely from their capacity to adhere to sites of injury and exert a variety of positive effects, including immunomodulation, angiogenesis promotion, neuroprotection, anti-apoptotic action, and inflammation suppression. Furthermore, they raise less ethical considerations and carry a lower risk of tumor formation than other stem cell types.^4^

Following transplantation, MSCs generated from human or rat donors can move specifically to injured brain areas, aiding in functional recovery. However, it seems doubtful that brain regeneration is fueled by the direct replacement of destroyed neurons *via* MSC differentiation, given only a small proportion of transplanted cells remain in the injured brain and an even smaller fraction differentiate into neural lineages. Their therapeutic activity is thus thought to be predominantly paracrine in nature, *via* the production of bioactive molecules that support endogenous healing pathways.

Despite these hopeful advancements, significant problems remain. These include inconsistencies in therapeutic outcomes, diversity in MSC sources and qualities, and the need for improved administration methods.^5–7^ Combining MSCs with biomaterials, such as scaffolds or hydrogels, may increase their survival, retention, and therapeutic efficacy.^1^ Continued research is critical to better understanding their *in vivo* mechanisms of action, standardizing protocols for treatment, and addressing safety concerns, especially for allogeneic use. MSC biology advances are broadening its applicability in personalized regenerative therapy, particularly through anti-inflammatory and immunoregulatory properties. As this science advances, well-designed clinical trials will be critical in converting experimental findings into viable, real-world treatments for neurodegenerative and neuroinflammatory disease.

Peptide-based approaches have been used to convert stem cells to neurons. Thermosensitive peptide-based CRP hydrogels^8^ imitate the spinal cord's regeneration environment, with injectable fluidity at low temperatures and a uniform 3D scaffold at body temperature to fill irregular spinal cord injury (SCI) sites. These hydrogels showed diverse therapeutic benefits, including shifting microglia/macrophage polarization towards an anti-inflammatory phenotype, lowering reactive astrogliosis, and increasing endogenous neural stem cell activation and neuronal differentiation at damage sites. The CRP hydrogels aided in total brain healing by promoting axonal regeneration, neuronal protection, and neural conduction restoration, which could be mediated by the PI3K/AKT/mTOR signaling pathway. These findings reveal new light on post-SCI inflammatory regulation, neural–glial connections, biomaterial-guided endogenous healing pathways and understanding of biomaterial-mediated neuronal regeneration. Furthermore, a unique therapeutic method was developed for the treatment of TBI that combines a neuroprotective self-assembling peptide hydrogel SLNAP with a small molecule NCM.^1^ NCM refers to a neuro-regenerative chemical modulator, a small chemical compound named SG-145,^2^ an imidazole-based small molecule that facilitates nerve repair and regeneration. Under physiological conditions, this hybrid multidomain peptide hydrogel transforms into an injectable, porous scaffold that entraps and gradually releases NCM at the injury site. The biodegradable SLNAP-NCM system displayed dual functionality, boosting hMSC trans-differentiation into functional neurons while also offering neuroprotection in brain damage models. Interestingly, this formulation increased both wound healing at the damage site and cognitive performance in CBI models. These findings indicate that this peptide hydrogel is both an outstanding neural cell culture platform and a promising transplanting scaffold for differentiated neurons. Again, free-standing silk fibroin films (SFFs) functionalized with laminin-derived YIGSR peptides to imitate the extracellular matrix that was used in neural tissue engineering applications.^9^ It was found that when covalently bonded peptide modifications (CL1-SFF and CL2-SFF) were paired with retinoic acid, they efficiently maintained hMSC stemness while allowing for on-demand neuronal differentiation. Notably, the CL2-SFF variation, which has an extra glycine spacer between the surface and the YIGSR peptide, outperformed other formulations in terms of encouraging hMSC development into mature neuron-like cells, even outperforming laminin protein-coated controls. This covalent modification technique outperformed physically adsorbed peptides in terms of long-term stability and differentiation efficiency, emphasizing the need of persistent surface functionalization. The success of CL2-SFF reveals that short laminin-mimetic peptides can substitute full-length laminin proteins for neuronal development. These engineered SFF scaffolds provide significant advantages, including simplified peptide synthesis, aqueous-based functionalization, and a special capability to either maintain stem cell pluripotency or direct neural differentiation when required, making them versatile platforms for regenerative medicine and neural tissue engineering. Next, a unique class of amyloid-inspired peptide hydrogels were developed from α-synuclein protein motifs.^10^ These gels may respond to heat or pH changes. These peptides form a cross-β-sheet-rich amyloid structure and self-assemble into a nanofibrous network that resembles the extracellular matrix. The modular design allows for simple sequence alterations to exploit the beneficial features of amyloids, making them intriguing candidates for cell-based treatments in neurodegenerative diseases. Notably, these hydrogels stimulate MSC adhesion, neuronal development, and effective engraftment in critical brain areas (substantia nigra and caudate putamen) in a Parkinson's disease mouse model.

At the same time, peptoid-based hydrogel gained attention for neuronal differentiation. Peptoids are sequence-defined oligomers of N-substituted glycine monomers (Fig. 1), easily synthesized using the solid-phase sub monomer synthesis process.^11^ The nitrogen atom in the backbone makes the differences in peptoid and peptide gels from each other. The side chains of peptoids are connected to nitrogen instead of carbon. This makes the backbone more flexible, less likely to break down by enzymes, and less likely to cause immunological reactions. These adjustments make peptoid gels more stable and adjustable than peptide gels. This versatile and efficient chemical allows for quick access to hundreds of possible monomers, opening a vast sequence space for exploration. Researchers from several domains can now customize peptoid sequences to address specific issues in biomedicine, nanoscience, and polymer science. Peptoids are widely regarded as more significant than peptides in many applications due to their higher stability, adaptability, and utility.^12^ Peptoids, unlike peptides, are very resistant to proteolysis because to their N-substituted backbone, which lacks the hydrogen-bonding amide proton, making them excellent for therapeutic and diagnostic applications. Peptoids improve cell membrane permeability by attaching side chains to nitrogen rather than α-carbon, which lowers hydrogen bonding and improves drug delivery. Their synthesis is also more efficient, using a modular sub monomer method that allows for the quick inclusion of various side chains, including non-natural groups, resulting in higher chemical diversity. Peptoids are less immunogenic than peptides, which reduces undesired immune responses in biological applications. It exhibited considerable potential as antimicrobial agents, replicating host-defense peptides without toxicity, as well as protein mimics in nanotechnology, such as peptoid nanosheets and helix bundles. Furthermore, unlike peptides, peptoids are stable under extreme pH and temperature conditions, broadening their use in industrial and environmental contexts. While peptides are still preferred for natural biological interactions that necessitate precise hydrogen bonding, peptoids excel in synthetic and manufactured environments.

**Fig. 1:**
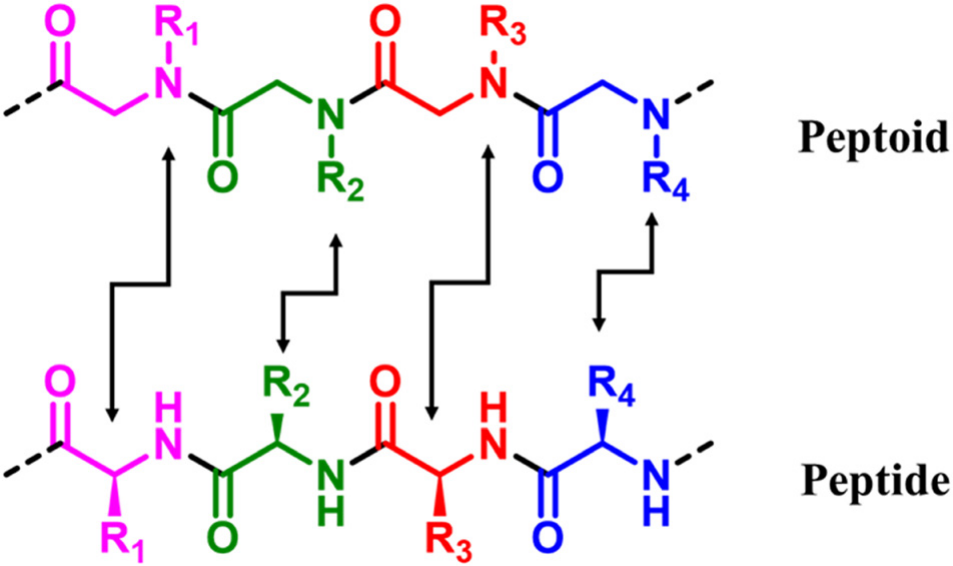
The structural differences between a peptide and peptoid.

Their programmable features make them useful for drug development, biomaterials, and biosensing. Overall, peptoids provide a strong alternative to peptides by combining durability, synthetic flexibility, and broad applicability, establishing them as an important tool in modern research and medicine.

Developing effective therapies for Alzheimer's disease (AD) a short tetrapeptoid called Ser–Leu–Lys–Pro (SLKP) was developed,^13^ which is derived from a neuroprotective amphibian brain-derived dodecapeptide. The SLKP peptoid demonstrated more neuroprotective benefits than its parent peptide, motivating further investigation using peptidomimetics. The SLKP peptoid showed high efficacy in suppressing amyloid-beta (Aβ) fibrillation, binding to tubulin, and stabilizing microtubule networks. The peptoid is serum stable, crosses the blood–brain barrier (BBB), and protects neurons from Aβ toxicity while increasing neurite outgrowth and neuronal health. This is the first report of a peptoid that has both neuroprotective and neuroregenerative capabilities, making it a prospective treatment candidate for Alzheimer's disease. Similarly, a short AI peptoid^14^ synthesized from the 30–34 hydrophobic region of the Aβ peptide. This molecule binds to the hydrophobic region of Aβ (17–21) and efficiently inhibits fibril formation. *In vitro* studies revealed that the peptoid also interacts with tubulin/microtubules, increasing their polymerization and stability. Furthermore, it inhibits acetylcholinesterase (AChE)-induced Aβ aggregation and provides neuroprotection in NGF-deprived PC12 neuron-like cells. Notably, the peptoid was serum stable and non-cytotoxic to primary rat cortical neurons, indicating its high potential compared to AI peptide in AD therapy.

Peptoids and peptides both have gelation properties, although their gel-forming capacities vary significantly due to structural and physicochemical variations. Peptoids, when coupled to peptides, can be used as excellent building blocks for molecular hydrogelators. Peptide stability was increased considerably by including peptoids into peptide architectures. Notably, two of the hydrogels had platelet-like morphologies, indicating the possibility of creating two-dimensional nanostructures capable of showing a high density of bioactive peptides. This structure may increase peptide activity *via* multivalent interactions.^15^

Peptoid-based gels are more resistant to enzymatic breakdowns and harsh circumstances (pH, temperature), making them effective when peptides fail. Recent developments in sequence design, such as amphiphilic and cyclic peptoids, have facilitated peptoid gelation. For validation, researchers develop a synthetic hydrogel system composed of poly (ethylene glycol) (PEG) macromers crosslinked with sequence-defined peptoids.^16^ They precisely manage hydrogel mechanics by changing peptoid structure and sequence, noting that helical peptoids increase the shear storage modulus in a way like helical peptide crosslinkers. The hydrogels have outstanding hydrolytic and enzymatic stability due to the N-substituted backbone of the peptoids. These findings not only increase the variety of peptide-based molecular hydrogelators but also offer a potential strategy for creating stable molecular hydrogels for use in controlled drug delivery and tissue engineering. Here, we discuss the potentiality of peptoid-based hydrogels to trans-differentiate hMSCs into functional neurons (Fig. 2). TBI sets off a chain reaction of damaging events in the brain that gets worse over time. The first big difficulty is that the BBB, which usually protects the brain, is broken. When this barrier is disrupted, fluids leak into the brain tissue, which makes it swell, and immune cells from the blood stream get into the damaged area. This not only hurts neurons directly, but it also causes a severe inflammatory reaction. Astrocytes and microglia, which are brain support cells, become very active. They don't totally fix the damage; instead, they release inflammatory molecules that speed up the death of neurons, which causes tissue loss and issues with memory, movement, or coordination. MSCs present a promising therapeutic approach due to their ability to traverse the compromised BBB post-transplantation and move towards the injured cerebral region. The MSCs are better supported when they are mixed with peptoid-based hydrogels because the hydrogel provides a sturdy structure and a good environment for cells to survive and work. This combination makes the brain more flexible, increases the connections between neurons, and helps with structural repair and functional recovery after a traumatic brain injury.

**Fig. 2:**
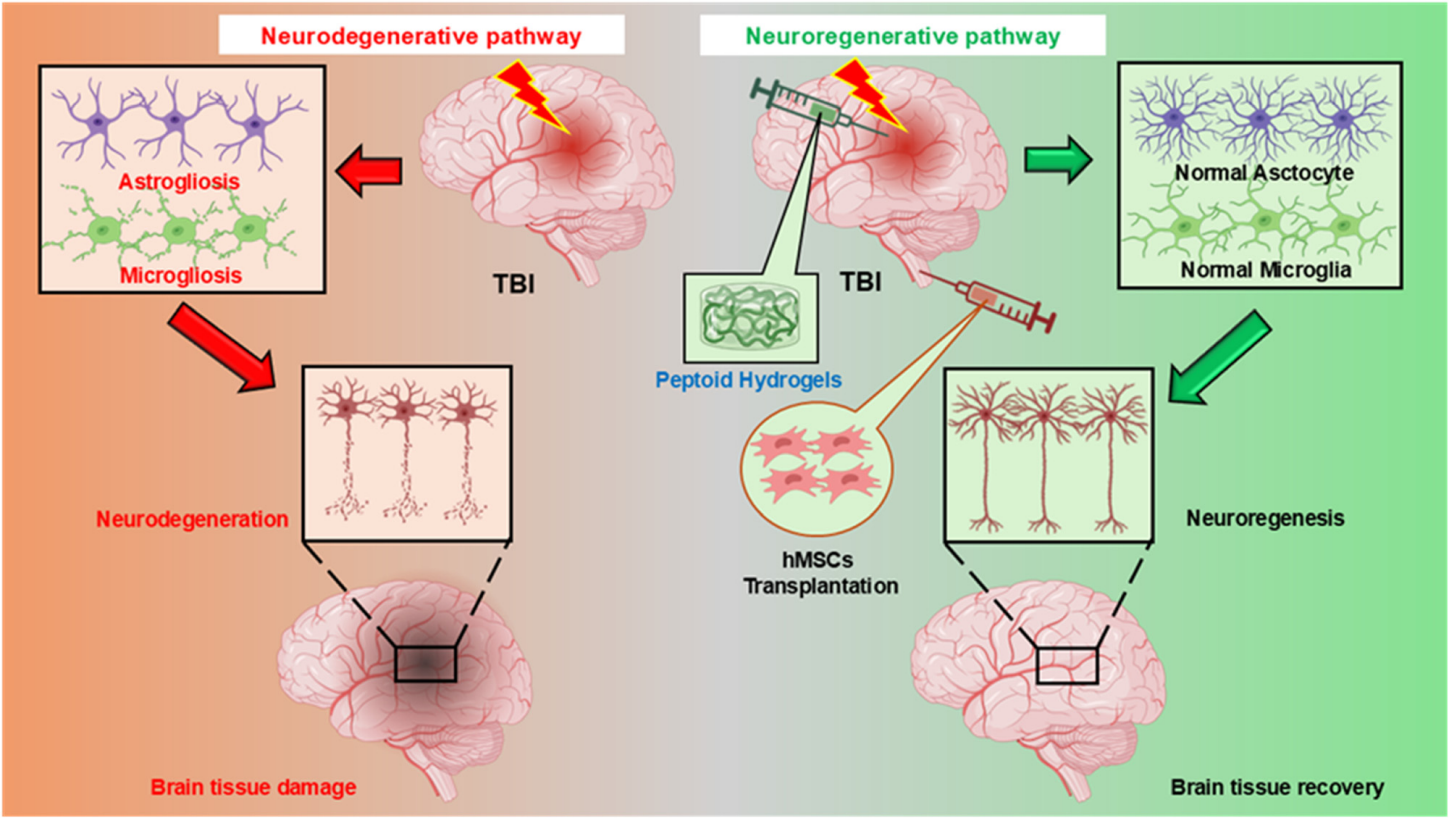
Potential mechanisms of neuroregeneration by peptoid-based hydrogel with hMSCs in TBI.

Future research should focus on maximizing the potential of peptoid-based hydrogels for brain repair. Efforts are required to optimize their structure, stiffness, and chemical properties to enhance the guidance of stem cells into functional neurons and promote their long-term viability. Development of injectable and responsive hydrogels for injury recovery. Simultaneously, additional animal studies are required to verify safety, integration, and stable recovery. These efforts suggest that peptoid hydrogels may serve as effective interventions for traumatic brain injury and various neurodegenerative diseases.

In summary, whereas many peptide-based hydrogels have been shown to promote the trans-differentiation of MSCs into functional neurons, there is a lack of available treatment techniques using peptoid-based hydrogels for neurodegenerative disorders. Future research should focus on optimizing peptoid biomaterial characteristics to improve neuronal differentiation efficiency under disease-relevant pathological conditions, while simultaneously promoting cell survival and functional stability. To obtain accurate delivery and increased therapeutic efficacy at the site of damage, the development of injectable, stimuli-responsive hydrogels with regulated and sustained drug release characteristics will be critical. Furthermore, extensive *in vivo* research is required to assess the long-term safety, functional integration, and regenerative capacity of neurons produced from peptoid hydrogels in disease models. With continuous multidisciplinary improvement, peptoid hydrogel-based therapies may have the potential to treat currently incurable neurodegenerative disease.

## Author contributions

Deviramma C. Yamanur and Pratikshya Paudel wrote the manuscript. Prabir Kumar Gharai conceived the idea and edited the manuscript.

## Conflicts of interest

The authors have no conflicts to declare.

## Abbreviations

TBI: Traumatic brain injury
MSCs: Mesenchymal stem cells
SCI: Spinal cord injury
SFFs: Silk fibroin films
AD: Alzheimer's disease
Aβ: Amyloid-beta
AChE: Acetylcholinesterase
PEG: Polyethylene glycol
BBB: Blood–brain barrier
NCM: Neuro-regenerative chemical modulator

## Acknowledgements

Deviramma C Yamanur thanks the IISER Tirupati. Pratikshya Paudel and Prabir Kumar Gharai thank Oklahoma State University.

## Data Availability

This opinion does not contain any primary research findings, software, or code, nor did it generate or analyse any new data.
